# Management of Prostate Cancer During COVID-19 Pandemic: Perspective From Urologists and Radiation Oncologists in COVID Dense Metro Detroit

**DOI:** 10.7759/cureus.9648

**Published:** 2020-08-10

**Authors:** Peter Domenig, Jacquelyn Booher, Benjamin Goldman, Jason Greenlee, Scott Sircus, Judith A Boura, Paul Chuba

**Affiliations:** 1 Urology, Ascension Macomb-Oakland Hospital, Warren, USA; 2 Radiation Oncology, Ascension Macomb-Oakland Hospital, Warren, USA

**Keywords:** prostate cancer, covid, metro detriot, urology, radiation oncology

## Abstract

Objective

To survey Urologists and Radiation Oncologists in Metropolitan Detroit regarding practice patterns in managing non-metastatic prostate cancer during the pandemic.

Methods

An online survey was created to capture the perspective of the impact the COVID-19 restrictions have on the management of prostate cancer by Urologists and Radiation Oncologists in the Detroit Metropolitan area.

Results

While most physicians felt that their facilities had adequate quantities of personal protective equipment (PPE), one in four offices reported that they did not have sufficient access to PPE. Urologists surveyed indicated that most of the low risk prostate cancer surgeries were cancelled and 56.2% had half or more of intermediate and high risk disease prostatectomies cancelled as well. Treatment options were then shifted towards either temporary surveillance or hormone therapy. Radiation Oncologists indicated that prostate cancer patients ready to start treatment were mostly delayed with temporary surveillance or hormone therapy depending on risk category (60% indicated they delayed low risk and favorable intermediate risk cases, 56% unfavorable intermediate risk cases, and 44% high risk cases). More than 80% of patients already undergoing treatment continued radiation.

Conclusion

In the setting of this pandemic, the management of prostate cancer has shifted to a much more conservative approach. While the response to the crisis has not been uniform, the majority of the practitioners followed newly established guidelines. The long-term outcomes of delays and deviations from standard treatment approaches will remain to be seen

## Introduction

Prostate cancer is a leading cause of cancer in men and commonly requires frequent patient-physician encounters for management. The stepwise process for localized prostate cancer begins with seeking a diagnosis with prostate-specific antigen (PSA) level, imaging and/or biopsy, and proceeds with deciding on a treatment plan [1]. Both diagnosis and treatment plans have recently been affected by the emergence of Coronavirus Disease 2019 (COVID-19). On March 10, 2020 the State of Michigan entered into a state of emergency after the first two positive cases of COVID-19 were detected [2]. The following day, COVID-19 was declared as a pandemic [3] by the World Health Organization (WHO). Metropolitan Detroit was among the hardest hit areas in the United States [2,3]. As of March 11, 2020 the WHO estimated more than 118,000 cases in 114 countries with 4,291 people having lost their lives [4-6]. At the time of this writing (May 16, 2020) there have been an estimated 4,374,690 cases worldwide with 294,394 deaths. Of these deaths, an estimated 87,315 deaths were reported in the USA and 4,825 deaths occurred in the State of Michigan, mainly clustered in the Detroit Metropolitan area [4-6].

A series of executive orders were issued by Michigan Governor Gretchen Whitmer beginning March 10, 2020 in an attempt to limit the spread of the disease [2,7,8]. As of March 21, 2020 medical providers were instructed to temporarily postpone “all non-essential procedures” defined as “not necessary to address a medical emergency or to preserve the health and safety of a patient” [8]. We endeavored to survey Urologists and Radiation Oncologists in Metropolitan Detroit regarding practice patterns in managing non-metastatic prostate cancer during the pandemic.

## Materials and methods

An online survey was created consisting of questions pertaining to the management of prostate cancer during the COVID-19 pandemic and was circulated via e-mail to Urologists and Radiation Oncologists in the Detroit Metropolitan Area (refer Appendices). There were 12 general questions answered both by Urologists and Radiation Oncologists with sixteen specialty-specific questions that were answered by Urologists and Radiation Oncologists separately. Dichotomous, multiple choice, and multiple answer questions were included. Demographics were captured within the general question section. The study was submitted to the institutional IRB and the survey was designated as exempt. 

Using the Google Forms™ platform, the survey was then emailed to 245 different Urologists and Radiation Oncologists in the Detroit Metropolitan area. The timeline to respond was three weeks. A reminder email was sent one week later to remind non-responders to complete the questionnaire. SAS® for Windows 9.4 (SAS Institute Inc., Cary, NC) was used for all analyses.

## Results

We received 46 responses from 21 (45%) Urologists and 25 (54%) Radiation Oncologists. This represents an overall response rate of 18.8%. The age of the physicians varied with the largest group belonging in the 51-60 years of age range (30.4%; 14/46). The majority of those surveyed had been in practice for > 10 years (82.6%; 38/46) which was evenly distributed from 10-30 years. Approximately half (47.8%; 22/46) of physicians were part of a household with 3-4 other people. All physicians indicated the ability to provide virtual office visits and 58.7% (27/46) were able to conduct more than half of prostate cancer patient encounters virtually. During routine examination, 80% (37/46) of respondents reported their patients were asked if they are COVID-19 positive. At least one COVID-19 diagnosis was noted at 65.2% (30/46) of facilities, of which approximately half were subsequently admitted to the hospital for this illness. This may be an underestimate since an additional 15.2% (7/46) responded that a patient or staff member may have been admitted. While most physicians felt that their facilities had adequate quantities of personal protective equipment (PPE), one in five offices felt that they did not have sufficient access to PPE. Although a majority of physicians maintained their subspecialty practices, 8.7% (4/46) of respondents had been asked to work or volunteered to work in another department of the hospital and 43.5% (20/46) indicated that their residents had been asked or volunteered to work in another department to help with the COVID-19 response. Those who responded ‘not-applicable’ were presumably not directly training residents in their practice. Both Urologists and Radiation Oncologists felt that they would return to normal clinical activities when a dramatic decline in COVID-19 cases occurs (24%; 11/46), when the hospital policy changes (37%; 17/46) or on a case-by-case basis (43.5%; 20/46).

With respect to Urologist’s practices for prostate biopsy, after the implementation of the stay at home executive order, 71.4% (15/21) of Urologists were still performing prostate biopsies in the office. There was no specific PSA cutoff for proceeding with biopsy for 20% (3/15) of Urologists, however, 40% (6/15) only performed biopsies for PSA values greater than 4, and 40% (6/15) would proceed for PSA values greater than 10. A significant majority felt that prostate MRI was an alternative to avoid or delay a repeat biopsy. Novel biomarkers for screening were not felt to have an increased value during the COVID-19 pandemic prior to considering biopsy for 57.1% (12/21) of Urologists [9,10]. Most responded that they would proceed with prostate biopsy after a COVID-19 positive patient has been without symptoms for 2-4 weeks. 

**Figure 1:**
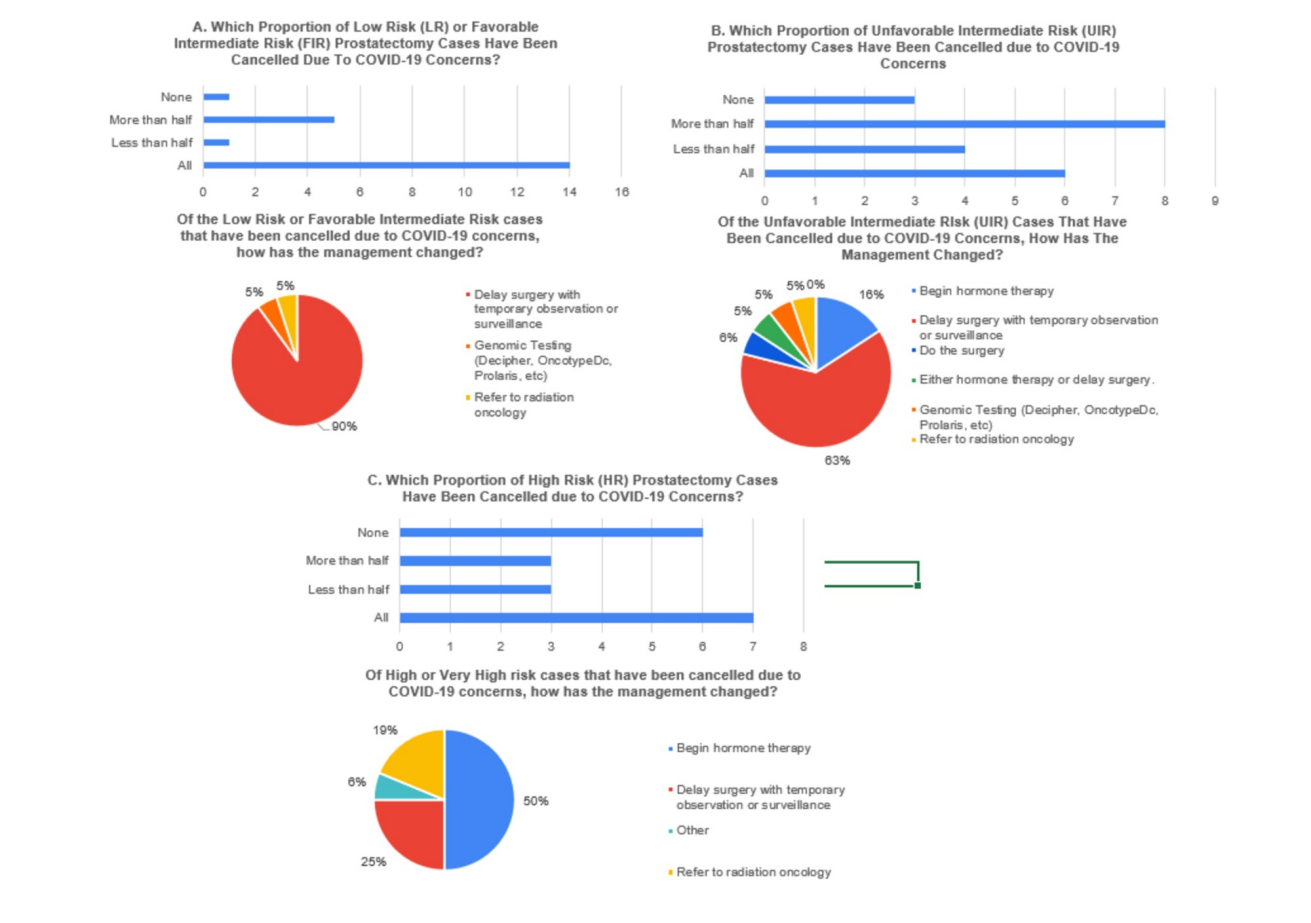
(A-C) Proportion of Cancelled Prostatectomy Cases and Management Changes

Responses from Urologists regarding prostatectomies are illustrated in Figure 1. A total of 66.7% (14/21) of respondents indicated that all low risk and favorable intermediate risk prostatectomy cases were cancelled and in nearly all of these cases the treatment plan chosen was to delay surgery with temporary observation or surveillance (90%; 18/20) (Figure 1A). 28.6% (6/21) of Urologists planning surgery for unfavorable intermediate risk prostate cases indicated they cancelled all cases and an additional 38.1% (8/21) responded they cancelled at least half of these cases (Figure 1B). For those cancelled, temporary observation or surveillance was preferred. With respect to high risk disease, 36.8% (7/19) indicated they cancelled all of these cases and an additional 15.8% (3/19) indicated they cancelled more than half (Figure 1C). In lieu of surgery, many of these high risk patients were started on hormone therapy (50%; 8/16) or referred to Radiation Oncology (18.8%; 3/16). 57.1% (12/21) did not feel that the delays in prostatectomy would change the surgical outcomes or that there would only be disease progression in unfavorable intermediate and/or high-risk patients.

Specific questions posed to Radiation Oncologists queried the use of PPE in the clinic, the ability for staff to work remotely, and the practice pattern for COVID positive patients. 11/25 respondents indicated that social distancing was implemented and 21/25 endorsed use of surgical masks for all staff. 100% of respondents indicated the ability to access treatment planning and/or Radiation Oncology system management software from home. There were 76% (19/25) of respondents who indicated that some or all of their dosimetrists and/or physicists were working from home. Of note, 28% (7/25) stated they had been asked to treat COVID-19 positive inpatients and 28% (7/25) had considered closing one or more treatment centers.

**Figure 2:**
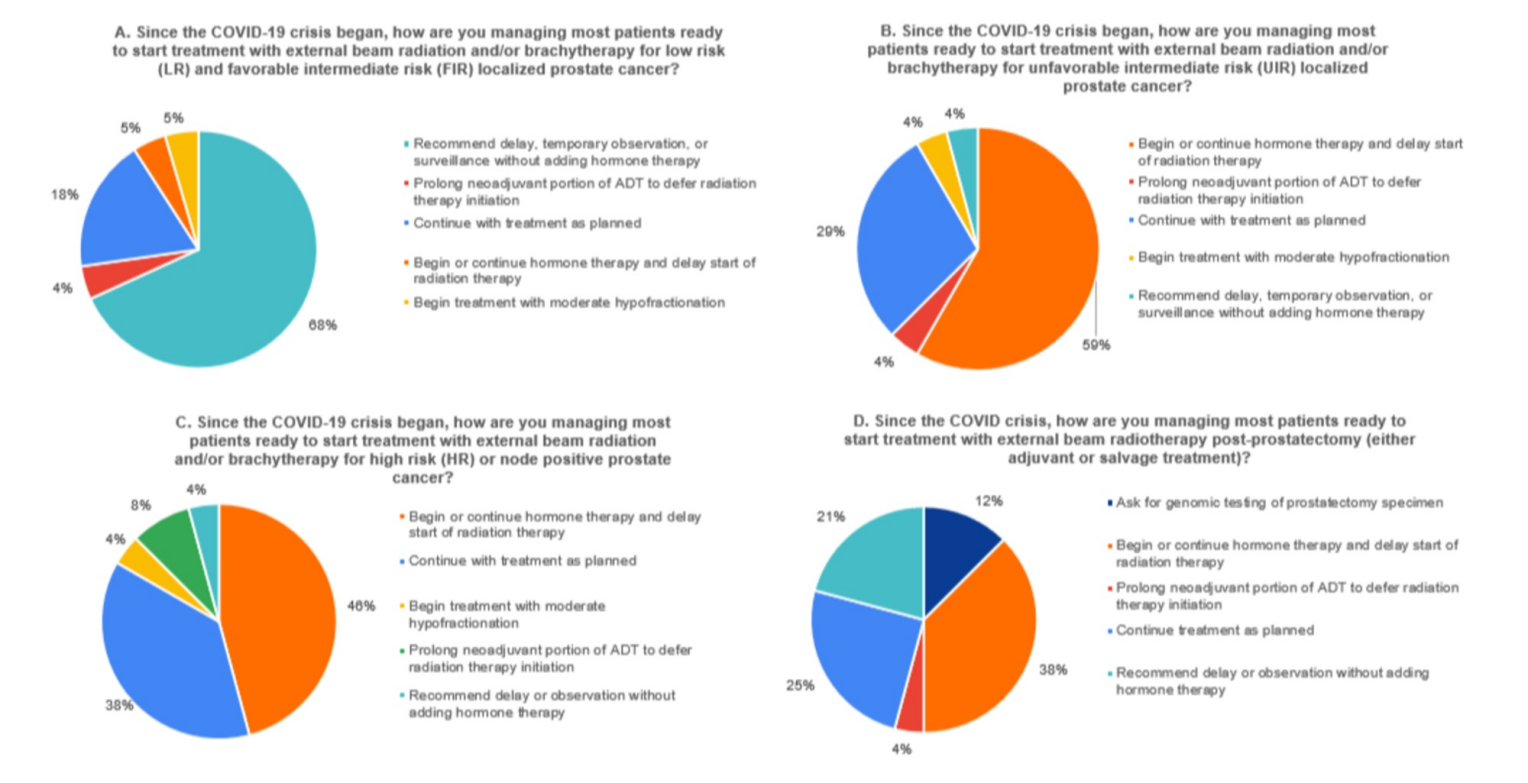
(A-D) Radiation Oncology Recommendations for Patients Ready to Start Treatment

Responses from Radiation Oncologists for patients ready to start treatment with external beam radiation and/or brachytherapy are illustrated in Figures 2A-2D. Radiation Oncologists mostly (68.2%; 15/22) recommended delay, temporary observation, or active surveillance for low-risk and favorable intermediate risk patients, but 18.2% (4/22) indicated they would continue with treatment as planned (Figure 2A). With respect to unfavorable intermediate risk cases, 58.3% (14/24) indicated they would delay treatment and begin or continue hormone therapy and 29.2% (7/24) continued with treatment as planned (Figure 2B). For high risk or node positive patients 45.8% (11/24) responded ‘begin or continue hormone therapy’, and 37.5% (9/24) responded ‘continue treatment as planned (Figure 2C). Finally, regarding patients recommended to start post-prostatectomy radiotherapy, 37.5% (9/24) would begin or continue hormone therapy and delay the start of radiation, and 25% (6/24) would continue with treatment as planned (Figure 2D). A few (3/25) indicated they would use genomic testing [11] to aid in decision making. The use of fiducial markers or rectal spacers in patients ready to start treatment (requiring an office procedure) was recommended by 33.3% (8/24) and an additional 45.8% (11/24) responded “maybe”. Physicians who did not recommend hormone therapy in some groups of patients cited a lack of proven benefit (41%; 9/22), potential clinical decline of the patient (27.3%; 6/22), and treatment toxicity (18.2%; 4/22). 

**Figure 3:**
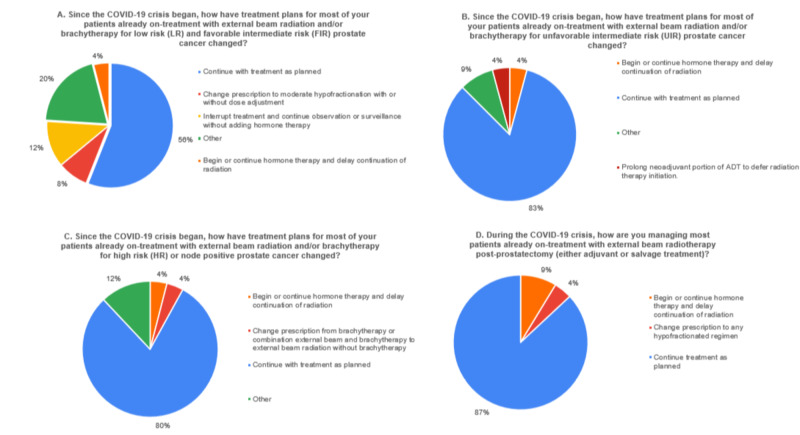
Radiation Oncology Recommendations for Patients Already On Treatment

Responses from Radiation Oncologists for patients already on-treatment with external beam radiation and/or brachytherapy are illustrated in Figures 3A-3D. With respect to treatment of patients already undergoing treatment, most (56%; 14/25) continued with treatment as planned for low risk and favorable intermediate risk cases (Figure 3A). A few physicians variably suggested that they would change prescriptions to use moderate hypofractionation, use SBRT methods, interrupt treatment, and continue on observation or surveillance, or begin hormone therapy. For patients already undergoing treatment for unfavorable intermediate risk 83.3% (20/24) of respondents indicated they continued treatment as planned (Figure 3B). One comment explained that in cases where brachytherapy was planned, external beam treatment would be delayed or started/continued on hormone therapy. Similarly, for patients with high risk or node positive prostate cancer, 80% (20/25) of those queried responded that they would continue treatment as planned (Figure 3C).

## Discussion

Until now, treatment decisions in prostate cancer have depended on the risk category and patient factors determining suitability for treatment [1]. Traditionally, this process has consisted of combined physician-patient decision making primarily involving Urologists and Radiation Oncologists and has required multiple office visits. The implementation of risk mitigation set forth by the State governor [2,7-8], to limit the spread of the disease, led to significant delays and barriers to the treatment of prostate cancer including the cancellation of elective surgeries. Given these unprecedented circumstances, Urologists and Radiation Oncologists were faced with new challenges in how to continue to manage patients with prostate cancer. Remarkably, up to 10% of the physicians surveyed indicated that they or their residents had been asked to work elsewhere in the hospital to respond to the crisis [12]. It is also notable that a relatively large percentage (26.1%; 12/46) indicated inadequate availability of personal protective equipment. The survey data presented here suggests significant heterogeneity in practice patterns for prostate cancer management during this crisis. 

Accepted treatment pathways for localized prostate cancer range from observation or active surveillance to prostatectomy, external beam radiation, or brachytherapy with or without hormone therapy or chemotherapy [1]. Some patients may receive postoperative radiation therapy. Thus, a multi-specialty approach is necessary for treatment of localized prostate cancer. Specific guidelines for patient care, and provider and practice information for use during the COVID crisis were rapidly proposed by the American College of Surgeons (ACS), the American Society for Clinical Oncology (ASCO) the American Society for Therapeutic Radiology and Oncology (ASTRO), the American Society of Anesthesiology (ASA) as well as a number of single institutions [13-19]. 

For Urologists, the cancellation of elective surgery by governmental edict helped to guide decision making. This is consistent with specific guidelines proposed by the ACS [13]. These define the acute phase with associated governmental bans on elective surgery, and recovery phase in which bans on surgery are lifted. The acute phase was in turn divided into three phases related to available resources and COVID trajectory for specific hospitals and/or regions. In the third, most severe phase, where all hospital resources are devoted to COVID, surgery is restricted to patients likely to have survivorship compromised if surgery is not performed immediately [13]. Most of the hospitals in the metro Detroit area, where our survey was distributed, fell into this category. By definition, this excludes performing elective surgery on those with prostate cancer. Aside from the risk of the spread of the virus among patients and hospital staff, the rationale for the cancellation of surgeries has been to minimize the use of scarce personal protective equipment for preferred use elsewhere in the hospital. Furthermore, in many cases cancelling surgery allowed use of the operating room space (including preoperative and recovery space) for intensive care patients and the use of equipment designed for general anesthesia instead for ventilation of COVID patients [16].

It is clear that many Urologists cancelled or delayed planned prostate cancer operations (Figure 1). The survey also indicates that with an increasing risk category, more cancer surgery was recommended. Many felt that there could be disease progression with delays for unfavorable intermediate risk and high risk cases (Figures 2A-2B). This perception seems to coincide with available literature reports describing treatment delay >6 months based on risk category [20,21]. However, a recent study suggested that delayed prostatectomy was not associated with adverse oncologic outcomes for intermediate or high risk disease [20,21]. A sum of 94.4% of respondents indicated they cancelled more than half of low and favorable intermediate risk prostate surgeries, 72% more than half of unfavorable intermediate risk cases, and 56.2% more than half of high risk cases (Figure 1). 

For Radiation Oncology prostate cancer decision making, specific guidelines were made available [17-19], however, decision making has largely been left up to the individual health systems, unaffiliated centers, and individual physicians. A widely used framework of recommendations specific for prostate cancer suggested that treatment could be “avoided or delayed until safe for very low, low, and favorable intermediate-risk disease” [17]. For unfavorable intermediate-risk, high risk, clinical node positive, recurrence post-surgery, oligometastatic, and low-volume M1 disease, the recommendation was for “neoadjuvant hormone therapy for 4-8 months as necessary”. “Ultrahypofractionation was preferred for localized, oligometastatic, and low volume M1, and moderate hypofractionation was preferred for post-prostatectomy and clinical node positive disease [17].” 

Based on the survey, it is clear that in Radiation Oncology, prostate cancer patients already undergoing treatment, mostly continued as planned depending on risk category (Figure 3). 56% of respondents indicated they continued low risk and favorable intermediate risk cases and 80% indicated they continued unfavorable intermediate risk and high risk cases. With respect to decision making in patients ready to start treatment, most (68%) indicated they would delay (temporary observation or active surveillance) low risk and favorable intermediate risk patients (Figure 2A). These percentages were lower for unfavorable intermediate risk (4%) and node positive or high risk cases (4%) (Figures 3B-3C). 

Advances in telemedicine in the USA [12] and worldwide [22] seem to have been linked to the response to COVID-19. The survey data show that essentially all of the physician practices represented, whether hospital based or independent, have moved towards virtual meetings with patients. To this end, certain regulations have been relaxed by governmental agencies. For example, the Centers for Medicare and Medicaid Services (CMS) broadened access to Medicare telehealth services on a temporary and emergency basis as part of the Coronavirus Preparedness and Response Supplemental Appropriations Act [23]. It appears that long-lasting solutions to billing and privacy issues related to virtual medicine are evolving rapidly. For Radiation Oncology specifically, it is interesting that a large proportion have the ability to access treatment planning software and system management software from home. Dosimetry and physics staff may increasingly be performing many of their traditional tasks remotely. 

Drawbacks of the current survey include the small numbers of respondents and the short time period in which the survey had to be assembled. The low response rate is a large drawback which might limit the reliability of the sample, however, those who did not respond probably would have answered in a similar manner. Smaller numbers for responses in the specific Urology and Radiation Oncology questions may be associated with under-sampling and statistical variability. Still, the data serve to point out considerable heterogeneity in the response to the crisis among those who participated. 

At the time of this writing, COVID-19 statistics for Michigan and for the Detroit Metropolitan Area are rapidly improving both in terms of new diagnoses and deaths. As the COVID-19 crisis continues and the curve flattens, this decision-making is being revised. Currently, in Michigan, the ‘stay at home’ order has been extended to May 29, 2020 [8]. In anticipation of re-opening, the classification of ‘time-sensitive’ surgery, which will include cancer surgeries such as prostatectomies, will likely affect treatment decisions in the near future [24]. A prioritization review team has been assembled and essential procedures important to the health of the patients over the coming months will resume [24]. We anticipate a large back-log of cases and short-term bottle-necks in scheduling.

## Conclusions

The COVID-19 pandemic has forced our health systems to adapt quickly to such an unprecedented challenge. Due to such a rapid spike in the demand for medical personnel, nearly 10% of established Urologists and Radiation Oncologists were asked to work in another department to help combat the pandemic. The survey presented here shows how the implementation of PPE and deployment of telemedicine encounters have quickly become commonplace in both Urology and Radiation Oncology practices. The data also indicates significant heterogeneity in the way individual physicians approached changes in the management of prostate cancer during the crisis. This data serves as a starting point for how clinicians are feeling with a shift to a more conservative approach in prostate cancer, deviating from the established guidelines and recommendations for the care of prostate cancer patients. Although there are many questions about how the COVID-19 pandemic will impact our medical community, it will also serve as an opportunity to explore the impact on prostate cancer outcomes.

## Appendices

Starting Questions

1.    Will you be asking your patients if they were/are COVID-19 positive during routine examinations?

2.    Has any patient or staff member in your facility been diagnosed clinically or tested positive for COVID-19 disease?

3.    Has any patient or staff member in your facility been admitted to the hospital for COVID-19 illness?

4.    In your opinion, do staff members at your facilities have access to adequate quantities of personal protective equipment (PPE)?

5.    What is your age?

6.    How many persons are there in your household (currently)

7.    How many years have you been practicing your specialty?

8.    Does your practice have the ability for virtual office visits?

9.    What portion of prostate cancer patient encounters have you been able to perform virtually?

10.  What portion of ALL patient encounters have you been able to perform virtually?

11. Have you been asked to work (or volunteered to work) in another department of the hospital because of the COVID-19?

12. Have your residents been asked to work (or volunteered to work) in another department of the hospital because of the COVID-19?

13. Are you a Urologist or Radiation Oncologist?

Urologist Questions

1.    Since March 24th, 2020 when the Governor of Michigan instituted a lock down, have you been performing prostate biopsies in office?

2.    If yes, is there a PSA cutoff for performing the biopsy?

3.    Would you consider MRI as an alternative to repeat biopsy during the COVID-19 pandemic to help avoid or delay re-biopsy?

4.    Would you consider novel biomarkers (4k score, SelectMDx, etc) to have an increased value during the COVID pandemic prior to considering biopsy?

5.    How long will you wait to biopsy a patient who was diagnosed as COVID-19 positive?

6.    Which proportion of Low Risk (LR) or Favorable Intermediate Risk (FIR) prostatectomy cases have been cancelled due to COVID-19 concerns?

7.    Of the Low Risk or Favorable Intermediate Risk cases that have been cancelled due to COVID-19 concerns, how has the management changed?

8.    Which proportion of Unfavorable Intermediate Risk (UIR) prostatectomy cases have been cancelled due to COVID-19 concerns

9.    Of the Unfavorable Intermediate RIsk (UIR) cases that have been cancelled due to COVID-19 concerns, how has the management changed?

10. Which proportion of High Risk (HR) prostatectomy cases have been cancelled due to COVID-19 concerns?

11. Of High or Very High risk cases that have been cancelled due to COVID-19 concerns, how has the management changed?

12. Will a patient's prior COVID-19 status change your operative approach for planned prostatectomies?

13. How long will you wait to perform a prostatectomy on a patient that was COVID-19 positive?

14. How do you feel that a delay in prostatectomy will change the surgical outcomes?

15. Do you agree that prostatectomy should be classified as elective surgery?

16. At what point in time do you anticipate resuming normal clinical activities

Radiation Oncology Questions

1.    Which anti-COVID-19 precautions are Radiation Oncology staff using:

2.    Do you have the ability to access treatment planning and/or the Radation Oncology system management software from home?

3.    Are some or all of your dosimetrists and/or physicists working from home?

4.    Has your facility been asked to treat COVID-19 positive inpatients?

5.    Have you considered closing one or more treatment centers?

6.    Since the COVID-19 crisis began, how have treatment plans for most of your patients already on-treatment with external beam radiation and/or brachytherapy for low risk (LR) and favorable intermediate risk (FIR) prostate cancer changed?

7.    Since the COVID-19 crisis began, how have treatment plans for most of your patients already on-treatment with external beam radiation and/or brachytherapy for unfavorable intermediate risk (UIR) prostate cancer changed?

8.    Since the COVID-19 crisis began, how have treatment plans for most of your patients already on-treatment with external beam radiation and/or brachytherapy for high risk (HR) or node positive prostate cancer changed?

9.    During the COVID-19 crisis, how are you managing most patients already on-treatment with external beam radiotherapy post-prostatectomy (either adjuvant or salvage treatment)?

10. Since the COVID-19 crisis began, how are you managing most patients ready to start treatment with external beam radiation and/or brachytherapy for low risk (LR) and favorable intermediate risk (FIR) localized prostate cancer?

11. Since the COVID-19 crisis began, how are you managing most patients ready to start treatment with external beam radiation and/or brachytherapy for unfavorable intermediate risk (UIR) localized prostate cancer?

12. Since the COVID-19 crisis began, how are you managing most patients ready to start treatment with external beam radiation and/or brachytherapy for high risk (HR) or node positive prostate cancer?

13. Since the COVID crisis, how are you managing most patients ready to start treatment with external beam radiotherapy post-prostatectomy (either adjuvant or salvage treatment)?

14. At what point in time do you anticipate resuming normal clinical activities

15. If you have indicated that you would continue planned treatments for some patients ready to start treatment, would you still recommend the use of fiducial markers or rectal spacers

16. If you are not recommending hormone therapies in some groups of patients, is this because:
